# Mortality Outcome Post-MI after PCI and CABG Interventions

**DOI:** 10.5334/gh.1556

**Published:** 2026-06-10

**Authors:** Mina Muayad Alwan Al-Naqdi, Mohammed Lateef Mohammed Alkhammasi, Bassam Muayad Alwan Al-Naqdi

**Affiliations:** 1 Al-Mustansiriyah Primary Healthcare Center, Baghdad, Iraq; 2 Al-Shaheed Al-Sadr General Hospital, Baghdad, Iraq; 3 Al Sheikh Zayed General Hospital, Baghdad, Iraq

**Keywords:** MI, PCI, CABG, mortality rate, survival analysis, Cox regression

## Abstract

**Background and Aim:**

Worldwide, heart-related conditions, including myocardial infarction (MI), persist as the leading cause of morbidity and mortality. The aim is to compare three-year mortality outcomes and identify causes of death among post-MI patients who received either percutaneous coronary intervention (PCI) or coronary artery bypass grafting (CABG).

**Methods:**

This registry-based retrospective cohort with follow-up study analyzed data from 3,542 PCI and 3,244 CABG patients treated post-MI between 2020 and 2024 in two hospitals in Baghdad. Kaplan–Meier curves (log-rank test) were used for unadjusted comparison. Cox proportional hazards regression was employed to compare three-year all-cause mortality, adjusting for baseline demographic and clinical covariates.

**Results:**

Baseline characteristics differed significantly, with CABG patients being older and having a higher prevalence of certain risk factors. Crude observed mortality during available follow-up was 7.99% for PCI and 11.19% for CABG; Kaplan–Meier analysis showed significantly different unadjusted survival distributions by log-rank test. However, after adjusting for baseline covariates, there was no significant difference in the hazard of three-year all-cause mortality between patients undergoing CABG and PCI (adjusted hazard ratio [aHR] = 1.12, 95% CI = 0.93–1.35, p = 0.280). Significant independent predictors of mortality included age (aHR = 1.04 per year), diabetes mellitus (aHR = 1.45), and renal complications (aHR = 1.70). Non-cardiovascular causes accounted for the majority of deaths in both groups (56.06% post-PCI, 50.11% post-CABG).

**Conclusion:**

In this observational cohort, adjusted three-year mortality was not significantly different between PCI and CABG, with non-cardiac causes accounting for the largest proportion of deaths. Due to potential confounding by indication, these findings represent observational associations rather than clinical equivalence. Both remain vital revascularization strategies, with selection guided by individualized heart-team assessment.

## Graphical Abstract

**Figure:**
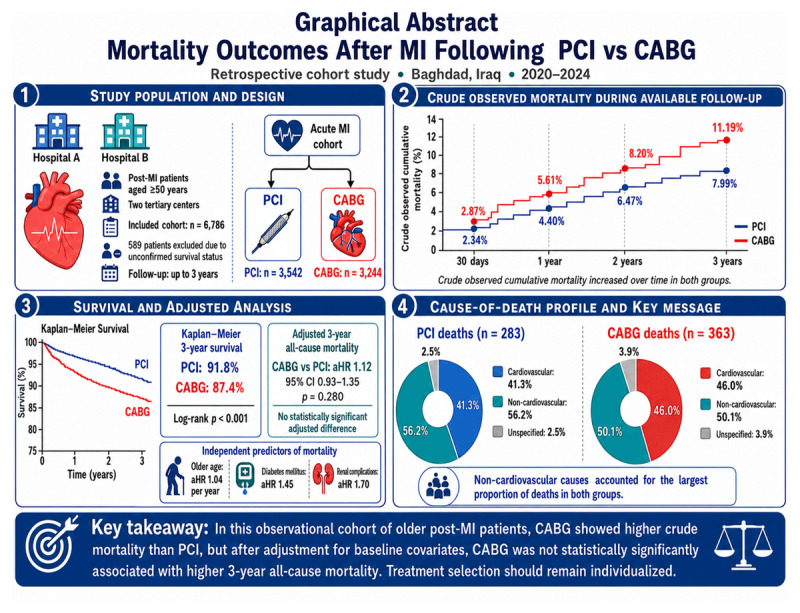


## Introduction

Globally, cardiovascular diseases, myocardial infarction (MI) in particular, continue to be the primary cause of morbidity and death (1). Effective revascularization techniques are necessary because managing post-MI patients remains difficult despite advances in medical therapies. Percutaneous coronary intervention (PCI) and coronary artery bypass grafting (CABG) are two commonly used procedures in this context, each with unique advantages and concerns (2).

MI is defined as acute myocardial injury with clinical evidence of myocardial ischemia, most commonly due to atherothrombotic coronary artery occlusion, in line with the Fourth Universal Definition of MI. This definition requires a rise and/or fall in cardiac troponin values with at least one value above the 99th percentile upper reference limit, together with symptoms of ischemia, new ischemic ECG changes, development of pathological Q waves, imaging evidence of new loss of viable myocardium or new regional wall motion abnormality, or identification of a coronary thrombus (3). In clinical practice, this encompasses ST-segment elevation MI (STEMI), non-ST-segment elevation MI (NSTEMI), and selected type 2 MI presentations.

After MI, there is frequently a crucial period in the acute phase during which time prompt therapies are necessary to preserve heart function and avert further problems. Many earlier studies have indicated a low death rate following non-cardiac surgical treatments in individuals who have had prior PCI or CABG (4,5,6). Moreover, contemporary randomized trials and registry analyses have compared PCI and CABG in patients with multivessel coronary artery disease and in those with acute coronary syndromes, including left main and non-ST-elevation presentations, and have generally shown that CABG offers more durable protection against major adverse cardiovascular events at the cost of higher peri-procedural stroke, while PCI is less invasive but associated with more repeat revascularization (7,8,9,10). In pooled analyses of left main disease, long-term all-cause mortality has been broadly similar between PCI and CABG, but with heterogeneous results across subgroups and follow-up durations (11,12). Comprehending the long-term effects of these revascularization treatments is essential for doctors and patients to make well-informed decisions (13). However, several important gaps remain. First, most comparative PCI versus CABG studies in the context of acute coronary syndromes come from high-income countries and highly selected trial populations, with limited evidence from real-world cohorts in low- and middle-income settings such as Iraq. Second, relatively few studies have described medium-term (≈3-year) outcomes, including cause-specific mortality, among older post-MI patients undergoing revascularization in routine practice. Third, there are almost no data from Baghdad or the wider region on how contemporary PCI and CABG outcomes compare in unselected hospital populations.

Therefore, we aimed to compare three-year all-cause and cause-specific mortality after PCI versus CABG in a large cohort of older patients hospitalized with acute MI in two tertiary centers in Baghdad between 2020 and 2024. We hypothesized that the adjusted association between revascularization strategy and long-term mortality would be attenuated after accounting for baseline risk profiles.

## Patients and Methods

### Study design

This study compares and examines three-year mortality outcomes in post-MI patients treated with either CABG or PCI.

We conducted a hospital-based, registry-derived retrospective cohort study of consecutive patients admitted with acute MI who subsequently underwent PCI or CABG. The ethics committees in the two participating hospitals approved the research protocol. Baseline clinical and procedural data were obtained retrospectively from electronic and paper medical records, while survival status and cause of death were ascertained through follow-up clinic visits and structured telephone contact up to three years after the index revascularization.

The index event was defined as the date of the PCI or CABG procedure performed during the index hospitalization for acute MI. The inclusion period spanned from January 1, 2020, to December 31, 2024. Each patient was followed up from the index date until death, completion of 36 months, or the study end date (December 31, 2024), whichever occurred first. Participants who remained alive and had not reached 36 months of follow-up by the database lock date were administratively right-censored at the time of their last contact (14,15).

### Study population

The study included registered acute MI (STEMI, NSTEMI, or other acute MI types) patients aged ≥50 years who underwent PCI or CABG during the index hospitalization from 2020 to 2024 in two hospitals in Baghdad, Iraq. Acute MI was defined according to the Fourth Universal Definition of MI, and the cohort included patients admitted with STEMI, NSTEMI, or other acute MI types as documented by the treating cardiologist and ECG findings. Only patients who underwent PCI or CABG as a primary intervention were included. Patients with previous CABG, those who did not undergo coronary revascularization, and those with missing key baseline variables or unknown vital status were excluded. We excluded 589 patients who declined research participation when contacted for follow-up, as their survival status could not be confirmed. Regarding clinical selection, the study utilized an ‘all-comers’ design to reflect real-world clinical practice. Therefore, patients were not excluded based on the extent of coronary artery disease (e.g., multivessel or left main disease), left ventricular ejection fraction (LVEF), or specific comorbidities such as diabetes mellitus or renal insufficiency. These clinical characteristics were recorded at baseline and utilized as covariates in the multivariable regression analysis to adjust for confounding factors.

In both hospitals, PCI and CABG were performed as part of the in-hospital management of the index acute MI. For this analysis, we included only procedures performed during the index hospitalization as the first coronary revascularization strategy after the qualifying MI (primary intervention). Patients who underwent elective PCI or CABG at a later admission, after initial conservative management, were not included. In STEMI, primary PCI was the default strategy when feasible, whereas urgent or early CABG was considered for patients with unsuitable coronary anatomy, mechanical complications, or failed PCI. In NSTEMI, an early invasive strategy with PCI or CABG was used according to coronary anatomy and heart-team discussion, in line with contemporary guidelines.

### Data collection and statistical analysis

For baseline data, demographic details (age, gender, and smoking status), medical history (cardiovascular comorbidities, hypertension, diabetes mellitus, prior heart complications, renal complications, and non-cardiac comorbidities, pulmonary, liver, cerebrovascular complications), type of MI (STEMI, non-STEMI, and others), and intervention type (PCI and CABG) were collected. Following the intervention, patients were followed up at regular intervals (e.g., 30 days, a year, two years, and three years).

Survival status and cause of death were recorded, categorized as either cardiac or non-cardiac based on available records. The survival duration was measured from the day of intervention until either the day of death or the end of the three-year follow-up.

Crude observed mortality at 30 days, one year, two years, and three years was summarized descriptively as cumulative observed deaths and proportions using baseline treatment-group denominators. These descriptive proportions were plotted separately and were not Kaplan–Meier estimates. Time-to-event survival functions were estimated using the Kaplan–Meier method, with participants censored at death, last known follow-up, completion of 36 months, or administrative study end, as appropriate. A log-rank test was employed to compare unadjusted survival distributions between the two groups. Baseline variables between PCI and CABG groups were compared by Chi-square tests or Fisher’s exact test for categorical variables and independent samples t-tests or Mann–Whitney U tests for continuous variables.

To assess the adjusted association between intervention type and three-year all-cause mortality, a Cox proportional hazards regression model was employed. The model used intervention type (CABG vs. PCI as the reference) as the primary predictor variable. To adjust for confounding by indication, a pre-specified, comprehensive variable selection strategy was utilized. All clinically relevant baseline variables (age, sex, smoking status, cardiovascular comorbidities, non-cardiovascular comorbidities, and MI type) were forced into the multivariable Cox proportional hazards model irrespective of their univariable statistical significance. No other unmeasured variables beyond those captured in the baseline registry were evaluated. The proportional hazards assumption was assessed using Schoenfeld residuals and found to be met for all covariates in the model. The output of the Cox model is presented as adjusted hazard ratios (aHRs) with 95% confidence intervals (CIs). Analyses were performed using SPSS software version 24 (IBM Corp., Armonk, NY, USA), with a p value of <0.05 indicating statistical significance.

Due to the rolling recruitment through 2024, follow-up durations varied. Thirty days, one year, two years, and three years were summarized descriptively as cumulative event counts and crude percentages. These descriptive percentages were not Kaplan–Meier estimates. Time-to-event survival functions were estimated using the Kaplan–Meier method, with participants censored at last known follow-up, and groups were compared using the log-rank test. Kaplan–Meier methods and Cox proportional hazards regression were used to account for censored observations and adjusted survival analysis, respectively (14).

Patients whose survival status could not be confirmed because they declined follow-up participation were excluded from the primary survival analysis. To assess potential selection bias, we summarized the distribution of these excluded patients by intended intervention group and compared their available baseline characteristics with those of included participants. Differences were described using standardized mean differences (SMDs). In addition, we performed deterministic sensitivity analyses to evaluate the possible effect of these exclusions on crude three-year mortality. Two extreme assumptions were examined: first, that none of the excluded patients died during follow-up; and second, that all excluded patients died during follow-up. These analyses were intended to quantify the potential direction and magnitude of selection bias and were not considered replacements for the primary time-to-event analysis.

## Results

The number of patients with completed follow-up interviews varied by time point due to the rolling recruitment and administrative censoring at the study end date. Table 1 details the number of patients who had the temporal opportunity to reach each follow-up landmark. Survival status was successfully ascertained for >96% of these eligible patients.

**Table 1:** Number of patients with potential follow-up duration available based on recruitment year (administrative censoring).


FOLLOW-UP TIME POINT	PCI GROUP (n)	CABG GROUP (n)

30 days	3,542	3,244

1 year	2,686	2,451

2 years	1,871	1,691

3 years	1,147	1,038


Abbreviations: CABG, coronary artery bypass grafting; PCI, percutaneous coronary intervention.

A total of 589 patients were excluded because their survival status could not be confirmed after they declined follow-up participation. Of these, 310 were in the PCI group, and 279 were in the CABG group (Table 2). Available baseline characteristics of excluded patients were broadly similar to those of included patients with respect to age, sex, diabetes, renal disease, and MI type, with no meaningful differences observed.

**Table 2:** Comparison of baseline characteristics between included and excluded patients, stratified by intervention group.


VARIABLE	INCLUDED PCI (n = 3,542)	EXCLUDED PCI (n = 310)	SMD	INCLUDED CABG (n = 3,244)	EXCLUDED CABG (n = 279)	SMD

Age (years), mean ± SD	64.2 ± 8.47	64.5 ± 8.60	<0.1	65.14 ± 9.38	65.4 ± 9.10	<0.1

Male sex, n (%)	1,982 (55.96%)	171 (55.16%)	<0.1	1,981 (61.07%)	167 (59.86%)	<0.1

Hypertension, n (%)	2,465 (69.59%)	216 (69.68%)	<0.1	2,106 (64.92%)	181 (64.87%)	<0.1

Diabetes mellitus, n (%)	944 (26.65%)	84 (27.10%)	<0.1	834 (25.71%)	73 (26.16%)	<0.1

Heart complications, n (%)	343 (9.68%)	30 (9.68%)	<0.1	180 (5.55%)	16 (5.73%)	<0.1

Renal complications, n (%)	217 (6.13%)	20 (6.45%)	<0.1	144 (4.44%)	13 (4.66%)	<0.1

Pulmonary complications, n (%)	455 (12.85%)	40 (12.90%)	<0.1	370 (11.41%)	32 (11.47%)	<0.1

Liver complications, n (%)	35 (0.99%)	3 (0.97%)	<0.1	27 (0.83%)	2 (0.72%)	<0.1

Cerebrovascular complications, n (%)	222 (6.27%)	19 (6.13%)	<0.1	163 (5.02%)	14 (5.02%)	<0.1

Current smoker, n (%)	2,293 (64.74%)	201 (64.84%)	<0.1	2,247 (69.27%)	193 (69.18%)	<0.1

Type of MI, n (%)			<0.1			<0.1

STEMI	1,245 (35.15%)	109 (35.16%)		1,120 (34.53%)	95 (34.05%)	

Non-STEMI	1,310 (36.98%)	115 (37.10%)		995 (30.67%)	86 (30.82%)	

Other	987 (27.87%)	86 (27.74%)		1,129 (34.80%)	98 (35.13%)	


Note: SMD < 0.1 is the standard threshold used. Abbreviations: CABG, coronary artery bypass grafting; MI, myocardial infarction; PCI, percutaneous coronary intervention; SMD, standardized mean differences; STEMI, ST-segment elevation MI.

In the primary analysis of included patients, crude observed three-year mortality was 7.99% in the PCI group and 11.19% in the CABG group. Under the sensitivity scenario, assuming that none of the excluded patients died, the corresponding crude three-year mortality estimates were 7.35% for PCI and 10.30% for CABG. Under the extreme scenario, assuming that all excluded patients died, the corresponding crude three-year mortality estimates were 15.39% for PCI and 18.22% for CABG. These sensitivity analyses suggest that the exclusion of patients with unconfirmed survival status does not materially affect the direction of the crude mortality comparison (with CABG mortality remaining higher than PCI in all scenarios), but it should be considered when interpreting the overall magnitude of mortality.

Between 2020 and 2024, 3,542 patients underwent PCI and 3,244 underwent CABG. The mean age was significantly higher in the CABG group than in the PCI group (65.14 ± 9.38 vs. 64.20 ± 8.47 years, p < 0.001), and the CABG group had a higher proportion of male patients (61.07% vs. 55.96%, p < 0.001) and current smokers (69.27% vs. 64.74%, p < 0.001). Hypertension was common in both groups but was more frequent in the PCI group (69.59% vs. 64.92%, p < 0.001). The PCI group also had higher proportions of heart complications (9.68% vs. 5.55%, p < 0.001), renal complications (6.13% vs. 4.44%, p = 0.002), and cerebrovascular complications (6.27% vs. 5.02%, p = 0.031). Diabetes mellitus, pulmonary complications, and liver complications did not differ significantly between groups. MI type distribution differed significantly between PCI and CABG groups (p < 0.001), with a higher proportion of non-STEMI in the PCI group and a higher proportion of other MI types in the CABG group. Table 3 presents the baseline demographic and clinical characteristics of included patients undergoing PCI versus CABG.

**Table 3:** Baseline demographic and clinical characteristics of included patients undergoing PCI versus CABG.


VARIABLE	PCI GROUP (n = 3,542)	CABG GROUP (n = 3,244)	P VALUE

Age (years), mean ± SD	64.2 ± 8.47	65.14 ± 9.38	<0.001

Male sex, n (%)	1,982 (55.96%)	1,981 (61.07%)	<0.001

Hypertension, n (%)	2,465 (69.59%)	2,106 (64.92%)	<0.001

Diabetes mellitus, n (%)	944 (26.65%)	834 (25.71%)	0.393

Heart complications, n (%)	343 (9.68%)	180 (5.55%)	<0.001

Renal complications, n (%)	217 (6.13%)	144 (4.44%)	0.002

Pulmonary complications, n (%)	455 (12.85%)	370 (11.41%)	0.076

Liver complications, n (%)	35 (0.99%)	27 (0.83%)	0.585

Cerebrovascular complications, n (%)	222 (6.27%)	163 (5.02%)	0.031

Current smoker, n (%)	2,293 (64.74%)	2,247 (69.27%)	<0.001

Type of MI, n (%)			<0.001

STEMI	1,245 (35.15%)	1,120 (34.53%)	

Non-STEMI	1,310 (36.98%)	995 (30.67%)	

Other	987 (27.87%)	1,129 (34.80%)	


Abbreviations: CABG, coronary artery bypass grafting; MI, myocardial infarction; PCI, percutaneous coronary intervention; STEMI, ST-segment elevation MI.

**Figure 1:**
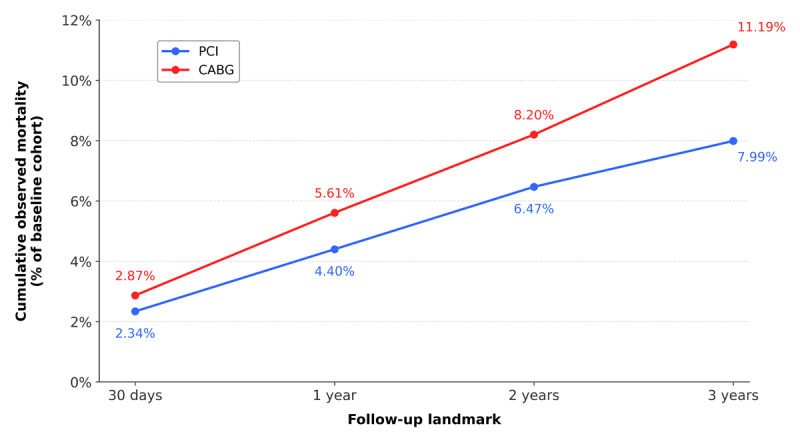
**Crude observed cumulative mortality during available follow-up in PCI and CABG groups.** Abbreviations: CABG, coronary artery bypass grafting; PCI, percutaneous coronary intervention.

During available follow-up, crude observed cumulative mortality increased across follow-up landmarks in both groups. At 30 days, observed mortality was 2.34% in the PCI group and 2.87% in the CABG group. By one year, mortality was 4.40% after PCI and 5.61% after CABG; by two years, it was 6.47% and 8.20%, respectively. At the final three-year landmark, crude observed mortality was 7.99% in the PCI group and 11.19% in the CABG group, corresponding to 283 and 363 deaths, respectively. Overall, 646 deaths were observed among included patients during available follow-up. These crude proportions are presented descriptively in Figure 1 and should not be interpreted as Kaplan–Meier estimates because follow-up availability varied due to rolling recruitment and administrative censoring.

**Figure 2:**
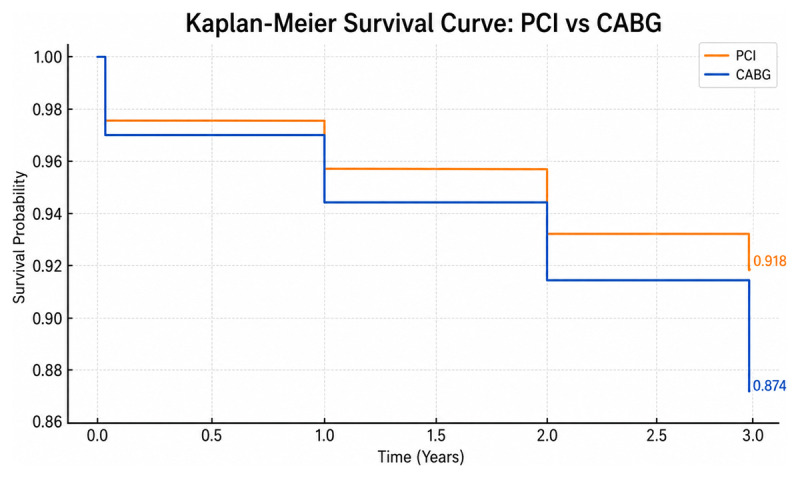
**Kaplan–Meier survival curves for PCI versus CABG over three years, log-rank test, p < 0.001**. Abbreviations: CABG, coronary artery bypass grafting; PCI, percutaneous coronary intervention.

Figure 2 displays the Kaplan–Meier survival curves, and the reported p values (<0.001) are derived from the log-rank test. Kaplan–Meier analysis showed higher unadjusted survival in the PCI group than in the CABG group. At three years, the Kaplan–Meier estimated survival probability was 91.8% for PCI and 87.4% for CABG (log-rank p < 0.001).

Table 4 summarizes the distribution of primary causes of death among patients who died during follow-up, using the total number of deaths within each intervention group as the denominator. Cardiovascular causes accounted for 41.3% of deaths after PCI and 46.0% after CABG, while non-cardiovascular causes accounted for 56.2% and 50.1%, respectively. Acute MI was the most frequent cardiovascular cause of death in both groups, followed by valve disease. Among non-cardiovascular causes, respiratory disease was the most common category in both the PCI and CABG groups. The cause of death was unspecified in 2.5% of PCI deaths and 3.9% of CABG deaths.

**Table 4:** Distribution of primary causes of death among patients who died during follow-up, stratified by intervention group.


CAUSE OF DEATH	PCI DEATHS, n (%)	CABG DEATHS, n (%)

**Cardiovascular causes**	**117 (41.3%)**	**167 (46.0%)**

Acute myocardial infarction	45 (15.9%)	65 (17.9%)

Valve disease	30 (10.6%)	45 (12.4%)

Heart failure	25 (8.8%)	19 (5.2%)

Cerebrovascular stroke	10 (3.5%)	10 (2.8%)

Chronic ischemic heart disease	7 (2.5%)	28 (7.7%)

**Non-cardiovascular causes**	**159 (56.2%)**	**182 (50.1%)**

Respiratory disease	65 (23.0%)	75 (20.7%)

Malignancy	35 (12.4%)	35 (9.6%)

Renal failure	20 (7.1%)	40 (11.0%)

Psychological factors/other	24 (8.4%)	17 (4.7%)

Liver failure	15 (5.3%)	15 (4.1%)

**Unspecified cause of death**	**7 (2.5%)**	**14 (3.9%)**

**Total deaths**	**283 (100.0%)**	**363 (100.0%)**


Note: Percentages are calculated using the total number of deaths within each intervention group as the denominator: PCI, n = 283 deaths; CABG, n = 363 deaths. Abbreviations: CABG, coronary artery bypass grafting; PCI, percutaneous coronary intervention.

After adjusting for baseline covariates, including age, gender, smoking status, hypertension, diabetes mellitus, and various complications (heart, renal, pulmonary, cerebrovascular) as well as the type of MI, the Cox proportional hazards model revealed no significant difference in the hazard of three-year all-cause mortality between patients who underwent CABG and those who received PCI (Table 5). The aHR was 1.12, with a 95% CI of 0.93–1.35 and a p value of 0.28.

**Table 5:** Multivariable Cox proportional hazards regression analysis for predictors of three-year all-cause mortality.


VARIABLE	aHR	95% CI	p VALUE

Intervention/PCI*			

CABG	1.12	0.93–1.35	0.280

Age	1.04	1.02–1.06	<0.001

Gender/female*			

Male	1.09	0.88–1.30	0.510

Current smoking status/No*			

Yes	1.15	0.95–1.40	0.150

Cardiovascular comorbidities			

Hypertension/no*	1.20	0.98–1.48	0.075

Diabetes mellitus/no*	1.45	1.20–1.75	0.002

History of heart complications/no*	1.10	0.90–1.34	0.350

History of renal complications/no*	1.70	1.35–2.15	<0.001

Non-cardiovascular comorbidities			

History of pulmonary complications/no*	1.08	0.85–1.37	0.520

History of liver complications/no*	0.97	0.65–1.45	0.880

History of cerebrovascular complications/no*	1.13	0.88–1.46	0.330

Type of myocardial infarction/others*			

STEMI	1.05	0.87–1.26	0.600

Non-STEMI	0.98	0.81–1.18	0.820


Abbreviations: aHR, adjusted hazard ratio; CABG, coronary artery bypass grafting; PCI, percutaneous coronary intervention; STEMI, ST-segment elevation MI. *Reference.

Significant independent predictors of three-year mortality identified in the adjusted model included age (aHR of 1.04 per year increase, 95% CI = 1.02–1.06, p < 0.001), the presence of diabetes mellitus (aHR = 1.45, 95% CI = 1.20–1.75, p = 0.002), and the presence of renal complications (aHR = 1.70, 95% CI = 1.35–2.15, p < 0.001). Gender and smoking status, however, were not found to be independent predictors of three-year mortality in the adjusted model (p > 0.05 for both).

## Discussion

This study represents one of the largest real-world comparisons of PCI and CABG outcomes in Baghdad, analyzing 6,786 post-MI patients over a three-year follow-up period. Crude observed mortality was higher in the CABG group than in the PCI group (11.19% vs. 7.99%). However, after adjustment for measured baseline covariates, CABG was not statistically significantly associated with a higher hazard of three-year all-cause mortality compared with PCI (aHR = 1.12, 95% CI = 0.93–1.35). These findings suggest that baseline differences between treatment groups may have contributed to the higher unadjusted mortality observed among CABG patients, although residual confounding by indication remains possible.

Our multivariable analysis identified advanced age, diabetes mellitus, and renal complications as the strongest independent predictors of long-term mortality. These findings are consistent with extensive literature establishing these variables as major determinants of prognosis in patients with coronary artery disease (7,16,17). Renal insufficiency, in particular, showed the highest hazard ratio in our cohort (aHR = 1.70), highlighting the systemic vulnerability of these patients regardless of the revascularization method chosen. Interestingly, while male gender and smoking status differed significantly at baseline, they were not independent predictors of mortality in the adjusted model, suggesting their effects may be mediated through the development of other comorbidities such as respiratory or cardiovascular disease.

As expected for cumulative outcomes, the number and crude proportion of observed deaths increased across longer follow-up landmarks in both treatment groups. These descriptive cumulative proportions should not be interpreted as evidence that the instantaneous hazard of death increased over time, as no time-varying hazard analysis was performed. In the Kaplan–Meier analysis, unadjusted survival was lower in the CABG group than in the PCI group by three years. However, after adjustment for measured baseline covariates, CABG was not statistically significantly associated with higher three-year all-cause mortality.

A pivotal finding of this study is the profile of cause-specific mortality. Contrary to older trials where cardiac causes of death typically predominated (17,18,19,20,21,22,23), our results showed that non-cardiovascular causes accounted for the majority of deaths in both the PCI (56.06%) and CABG (50.11%) groups. The lower proportions of cardiac cause of death in this cohort might be due to differences in patient risk profiles (i.e., older population in the US cohorts, possibly making them more vulnerable to non-cardiac reasons such as pulmonary diseases). This result aligns with the study (19) that had non-cardiac reasons of death higher than cardiac after PCI, and similarly has an older population. This also aligns with recent trends in high-income countries, where improvements in secondary prevention (e.g., potent antiplatelet therapy, statins, and heart failure management) have successfully reduced cardiac mortality, thereby unmasking non-cardiac competing risks such as respiratory disease and malignancies (19,20). In our cohort, respiratory complications were the most frequent non-cardiac cause of death. This may reflect the high prevalence of smoking in our population and suggests that long-term survival after revascularization in Iraq is increasingly dependent on the management of non-cardiac comorbidities rather than recurrent ischemic events alone. The unspecified causes of death in our study were low compared to other studies, where study (24) reported a 25% undetermined cause of death after PCI.

Overall, the distribution of causes of death showed overlapping patterns between the PCI and CABG groups. Our adjusted analysis, finding no significant difference in three-year mortality, supports the conclusion that both PCI and CABG are viable revascularization options for older patients post-MI. The decision-making process should therefore be highly individualized, considering coronary anatomy, patient comorbidities, procedural risks, and patient preferences, alongside diligent management of both cardiac and non-cardiac risk factors in the long term.

Our findings add crucial data to the existing body of evidence, which is heavily skewed toward populations in North America and Western Europe. Because after adjustment for measured baseline covariates, CABG was not statistically significantly associated with a higher hazard of three-year all-cause mortality compared with PCI in a Middle Eastern all-comers setting, this study supports current international guidelines advocating for the heart-team approach. The decision between PCI and CABG should therefore not be driven by a presumed mortality difference, but rather by anatomical complexity (e.g., SYNTAX score), surgical risk, and patient preference (2,12).

The strengths of this study include its large sample size and the inclusion of an unselected real-world population, offering high external validity for clinical practice in the region. However, several limitations must be acknowledged. First, the observational design introduces the potential for residual confounding despite multivariable adjustment. Second, while we captured broad categories of death, specific causes were unavailable for a small percentage of patients. Third, we did not have access to angiographic complexity scores (such as the SYNTAX score) to further stratify the comparative efficacy of PCI versus CABG. Fourth, due to the observational nature of the study, treatment assignment was not randomized. Patients undergoing CABG were older and had more comorbidities, indicating potential confounding by indication. While we utilized multivariable Cox regression to adjust for these differences, the potential for unmeasured residual confounding remains. Moreover, the exclusion of 589 patients with unconfirmed survival status may have introduced selection bias if non-participation was related to prognosis. Although we compared available baseline characteristics between included and excluded patients and performed extreme-scenario sensitivity analyses, the true survival status of these excluded patients remains unknown. Therefore, the reported mortality estimates should be interpreted with caution. As the study was conducted in two major tertiary centers, results may not be generalizable to centers with lower procedural volumes. Finally, it is important to note that this study was not formally designed or powered as a non-inferiority or equivalence trial with a pre-specified equivalence margin. Therefore, the absence of statistical significance in our multivariable model does not constitute definitive evidence of clinical equivalence between PCI and CABG.

## Conclusion

In this real-world observational cohort of older post-MI patients in Baghdad, cumulative mortality increased with longer follow-up in both PCI and CABG groups, as expected for cumulative survival outcomes. Crude observed three-year mortality was higher after CABG than after PCI; however, after adjustment for measured baseline covariates, CABG was not statistically significantly associated with higher three-year all-cause mortality compared with PCI. Non-cardiovascular causes accounted for the largest proportion of deaths among patients who died in both groups. Because treatment allocation was not randomized and residual confounding by indication remains possible, these findings should be interpreted as observational associations rather than evidence of causal equivalence between PCI and CABG. Treatment selection should remain individualized, considering coronary anatomy, procedural feasibility, comorbidity burden, surgical risk, and patient preference.

## Data Availability

Data are available upon reasonable request.

## References

[1] Ralapanawa U, Sivakanesan R. Epidemiology and the magnitude of coronary artery disease and acute coronary syndrome: A narrative review. J Epidemiol Glob Health. 2021;11:169–177. DOI: 10.2991/jegh.k.201217.001 33605111 PMC8242111

[2] Vervoort D, Sud M, Zeis TM, Haouzi AA, An KR, Rocha R, et al. Do the few dictate care for the many? Revascularization considerations that go beyond the guidelines. Can J Cardiol. 2024;40(2):275–289. DOI: 10.1016/j.cjca.2023.11.007 38181974

[3] Thygesen K, Alpert JS, Jaffe AS, Chaitman BR, Bax JJ, Morrow DA, et al. Fourth universal definition of myocardial infarction. Circulation. 2018;138:e618–e651. DOI: 10.1161/CIR.0000000000000617 30571511

[4] Hosseiny AD, Moloi S, Chandrasekhar J, Farshid A. Mortality pattern and cause of death in a long-term follow-up of patients with STEMI treated with primary PCI. Open Hear. 2016;3:e000405. DOI: 10.1136/openhrt-2016-000405 PMC4836287 27099764

[5] McFalls EO, Ward HB, Moritz TE. Coronary-artery revascularization before elective major vascular surgery. J Vasc Surg. 2005;41:733. DOI: 10.1056/NEJMoa041905

[6] Marquis-Gravel G, Dalgaard F, Jones AD, Lokhnygina Y, James SK, Harrington RA, et al. Post-discharge bleeding and mortality following acute coronary syndromes with or without PCI. J Am Coll Cardiol. 2020;76:162–171. DOI: 10.1016/j.jacc.2020.05.031 32646565

[7] Ramanathan K, Abel JG, Park JE, Fung A, Mathew V, Taylor CM, et al. Surgical versus percutaneous coronary revascularization in patients with diabetes and acute coronary syndromes. J Am Coll Cardiol. 2017;70:2995–3006. DOI: 10.1016/j.jacc.2017.10.029 29241487

[8] Zhang X-L, Zhu Q-Q, Yang J-J, Chen Y-H, Li Y, Zhu S-H, et al. Percutaneous intervention versus coronary artery bypass graft surgery in left main coronary artery stenosis: A systematic review and meta-analysis. BMC Med. 2017;15:84. DOI: 10.1186/s12916-017-0853-1 28427392 PMC5399381

[9] Shiomi H, et al. Comparison of percutaneous coronary intervention with coronary artery bypass grafting in unprotected left main coronary artery disease–5-year outcome from CREDO-Kyoto PCI/CABG Registry Cohort-2. Circ J. 2015;79:1282–1289. DOI: 10.1253/circj.CJ-15-0034 25818902

[10] Pyxaras SA, et al. Long-term clinical outcomes after percutaneous coronary intervention versus coronary artery bypass grafting for acute coronary syndrome from the DELTA registry: A multicentre registry evaluating percutaneous coronary intervention versus coronary artery bypass grafting for left main treatment. EuroIntervention. 2016;12:e623–e631. DOI: 10.4244/EIJV12I5A102 27497362

[11] Januszek R, et al. Long-term outcomes of percutaneous coronary interventions within coronary artery bypass grafts. Arch Med Sci. 2021;17:628–637. DOI: 10.5114/aoms.2018.75608 34025832 PMC8130480

[12] Gaba P, et al. Percutaneous coronary intervention vs coronary artery bypass graft surgery for left main disease in patients with and without acute coronary syndromes: A pooled analysis of 4 randomized clinical trials. JAMA Cardiol. 2023;8:631–639. DOI: 10.1001/jamacardio.2023.1177 37256598 PMC10233454

[13] Kok MM, von Birgelen C. Involving the patient’s perspective and preferences concerning coronary angiography and percutaneous coronary intervention. EuroIntervention. 2020;15:1228–1231. DOI: 10.4244/EIJV15I14A221 32044732

[14] Schober P, Vetter TR. Survival analysis and interpretation of time-to-event data: The tortoise and the hare. Anesth Analg. 2018;127:792–798. DOI: 10.1213/ANE.0000000000003653 30015653 PMC6110618

[15] Rich JT, et al. A practical guide to understanding Kaplan-Meier curves. Otolaryngol Neck Surg. 2010;143:331–336. DOI: 10.1016/j.otohns.2010.05.007 PMC3932959 20723767

[16] Radico F, et al. Determinants of long-term clinical outcomes in patients with angina but without obstructive coronary artery disease: A systematic review and meta-analysis. Eur Heart J. 2018;39:2135–2146. DOI: 10.1093/eurheartj/ehy185 29688324

[17] Pedersen F, et al. Short-and long-term cause of death in patients treated with primary PCI for STEMI. J Am Coll Cardiol. 2014;64:2101–2108. DOI: 10.1016/j.jacc.2014.08.037 25457398

[18] Fokkema ML, et al. Population trends in percutaneous coronary intervention: 20-year results from the SCAAR (Swedish Coronary Angiography and Angioplasty Registry). J Am Coll Cardiol. 2013;61:1222–1230. DOI: 10.1016/j.jacc.2013.01.007 23500325

[19] Spoon DB, et al. Trends in cause of death after percutaneous coronary intervention. Circulation. 2014;129:1286–1294. DOI: 10.1161/CIRCULATIONAHA.113.006518 24515993

[20] Tran DT, et al. Total and cause-specific mortality after percutaneous coronary intervention: Observations from the Alberta provincial project for outcome assessment in coronary heart disease registry. CJC Open. 2019;1:182–189. DOI: 10.1016/j.cjco.2019.05.003 32159105 PMC7063620

[21] Morice M-C, et al. Outcomes in patients with de novo left main disease treated with either percutaneous coronary intervention using paclitaxel-eluting stents or coronary artery bypass graft treatment in the synergy between percutaneous coronary intervention with TAXUS and C. Circulation. 2010;121:2645–2653. DOI: 10.1161/CIRCULATIONAHA.109.899211 20530001

[22] Tran DT, et al. Quality of acute myocardial infarction care in Canada: A 10-year review of 30-day in-hospital mortality and 30-day hospital readmission. Can J Cardiol. 2017;33:1319–1326. DOI: 10.1016/j.cjca.2017.06.014 28941611

[23] Tokushige A, et al. Incidence and outcome of surgical procedures after coronary artery bypass grafting compared with those after percutaneous coronary intervention: A report from the Coronary Revascularization Demonstrating Outcome Study in Kyoto PCI/CABG Registry Cohort-2. Circ Cardiovasc Interv. 2014;7:482–491. DOI: 10.1161/CIRCINTERVENTIONS.113.001056 25074253

[24] Stolker JM, et al. Mode of death after contemporary percutaneous coronary intervention: A report from the evaluation of drug eluting stents and ischemic events registry. Am Heart J. 2011;162:914–921. DOI: 10.1016/j.ahj.2011.08.014 22093209

